# Cellular Mechanisms of Sinus Node Dysfunction in Carriers of the *SCN5A*-E161K Mutation and Role of the H558R Polymorphism

**DOI:** 10.3389/fphys.2018.01795

**Published:** 2018-12-18

**Authors:** Ronald Wilders

**Affiliations:** Department of Medical Biology, Amsterdam University Medical Centers, Amsterdam, Netherlands

**Keywords:** computer simulations, electrophysiology, genetics, human, ion channel, sinus bradycardia, sinus node dysfunction, sodium current

## Abstract

**Background:** Carriers of the E161K mutation in the *SCN5A* gene, encoding the Na_V_1.5 pore-forming α-subunit of the ion channel carrying the fast sodium current (I_Na_), show sinus bradycardia and occasional exit block. Voltage clamp experiments in mammalian expression systems revealed a mutation-induced 2.5- to 4-fold reduction in I_Na_ peak current density as well as a +19 mV shift and reduced steepness of the steady-state activation curve. The highly common H558R polymorphism in Na_V_1.5 limits this shift to +13 mV, but also introduces a -10 mV shift in steady-state inactivation.

**Aim:** We assessed the cellular mechanism by which the E161K mutation causes sinus node dysfunction in heterozygous mutation carriers as well as the potential role of the H558R polymorphism.

**Methods:** We incorporated the mutation-induced changes in I_Na_ into the Fabbri-Severi model of a single human sinoatrial node cell and the Maleckar et al. human atrial cell model, and carried out simulations under control conditions and over a wide range of acetylcholine levels.

**Results:** In absence of the H558R polymorphism, the E161K mutation increased the basic cycle length of the sinoatrial node cell from 813 to 866 ms. In the simulated presence of 10 and 25 nM acetylcholine, basic cycle length increased from 1027 to 1131 and from 1448 to 1795 ms, respectively. The increase in cycle length was the result of a significant slowing of diastolic depolarization. The mutation-induced reduction in I_Na_ window current had reduced the contribution of the mutant component of I_Na_ to the net membrane current during diastolic depolarization to effectively zero. Highly similar results were obtained in presence of the H558R polymorphism. Atrial excitability was reduced, both in absence and presence of the H558R polymorphism, as reflected by an increase in threshold stimulus current and a concomitant decrease in capacitive current of the atrial cell.

**Conclusion:** We conclude that the experimentally identified mutation-induced changes in I_Na_ can explain the clinically observed sinus bradycardia and potentially the occasional exit block. Furthermore, we conclude that the common H558R polymorphism does not significantly alter the effects of the E161K mutation and can thus not explain the reduced penetrance of the E161K mutation.

## Introduction

The “fast sodium current” (I_Na_), which flows through Na_V_1.5 sodium channels, is a key player in the electrical activity of the human heart (Zimmer et al., 2014), where it is responsible for the fast >150 V/s upstroke of individual atrial and ventricular cardio-myocytes (Workman et al., 2001; O’Hara et al., 2011). The cardiac-specific Na_V_1.5 protein, encoded by the *SCN5A* gene, is the pore-forming α-subunit of the channel (Remme, 2013). I_Na_ has also been observed in patch-clamp recordings from isolated human sinoatrial (SA) node cells (Verkerk et al., 2009), in line with the observation by Chandler et al. (2009) that Na_V_1.5 is expressed in human SA nodal tissue. Accordingly, I_Na_ was included in the comprehensive computational model of a single human SA nodal cell that was recently developed by Fabbri et al. (2017) and is known as the Fabbri-Severi model.

Mutations in genes encoding ion channel-related proteins may result in inherited arrhythmia disorders, in particular the long QT syndrome (LQTS) and the Brugada syndrome (BrS). Gain-of-function and loss-of-function mutations in *SCN5A* underlie LQTS type 3 (LQT3) and BrS type 1 (BrS1), respectively (Brugada et al., 2018; Giudicessi et al., 2018; Wilde and Amin, 2018). Mutations in *SCN5A* may also lead to overlapping phenotypes (*SCN5A* overlap syndromes), where clinical characteristics of LQTS, BrS, and other arrhythmia syndromes, e.g., conduction disease and sinus node dysfunction, coexist in a single family or even in a single patient (Remme et al., 2008; Giudicessi et al., 2018). Sinus node dysfunction is a relatively common observation in both LQT3 and BrS1 patients (Hayashi et al., 2018; Wilders and Verkerk, 2018). It should be noted that the “gain of function” in case of LQT3 refers to the persistent I_Na_ that prolongs the ventricular action potential and thus causes QT interval prolongation. If an LQT3 mutation is associated with sinus bradycardia, this mutation shows a concomitant “loss of function” in the voltage range of diastolic depolarization (Wilders and Verkerk, 2018).

In 2005, Smits et al. reported the clinical and biophysical features of a novel sodium channel mutation, E161K, i.e., the replacement of the highly conserved acidic residue glutamic acid (E) with the basic residue lysine (K) at position 161 of the Na_V_1.5 protein (p.Glu161Lys; c.481G > A) (Smits et al., 2005). This loss-of-function mutation was identified in individuals of two non-related families. Affected patients had a complex clinical phenotype with symptoms of bradycardia, sinus node dysfunction, generalized conduction disease or BrS, or various combinations thereof. Sinus node dysfunction was observed in 8 out of 14 mutation carriers. Twenty-four-hour Holter recordings from 10 mutation carriers revealed that their absolute minimum heart rate, but not their maximum heart rate, was significantly lower compared to controls (39 ± 1 vs. 51 ± 0.6 beats/min, mean ± SEM). Furthermore, the incidence of signs of sinus node dysfunction in E161K mutation carriers was particularly high at night, when vagal tone is dominant.

Smits et al. (2005) assessed the biophysical effects of the E161K mutation by transfecting E161K or wild-type sodium channel α-subunit into tsA201 cells, together with wild-type β_1_-subunit. Voltage clamp experiments on the transfected cells revealed a 2.5-fold reduction in peak I_Na_ at -20 mV for E161K sodium channels compared to wild-type channels. Furthermore, the steady-state activation curve of the mutant channels showed a +11.9 mV shift of its half-maximal activation potential (V_1/2_) compared to wild-type (-30.7 ± 0.8 vs. -42.6 ± 1.4 mV, respectively). Also, the steepness of this curve was slightly reduced; its slope factor (k) amounted to 7.9 ± 0.3 vs. 6.7 ± 0.4 mV, respectively. Voltage dependence of steady-state inactivation, recovery from inactivation, and development of slow inactivation were not affected by the E161K mutation. Of note, these data were all obtained in H558 background, i.e., with histidine (H) at position 558 of the Na_V_1.5 protein.

Iwasa et al. (2000) were the first to report on the c.1673A > G single nucleotide polymorphism (“SNP”) in the *SCN5A* gene, which is responsible for the replacement of histidine (H) with arginine (R) at position 558 of the Na_V_1.5 protein (p.His558Arg or H558R), in relation to familial LQTS. Ackerman et al. (2004) showed that H558R is the most common polymorphism in *SCN5A* and that this variant is present in all four ethnic groups, albeit at a significantly lower prevalence in Asians. In blacks, whites, and Hispanics, the prevalence of R558 instead of H558 amounts to 20–30%, whereas this prevalence is near 10% among Asians (Ackerman et al., 2004). Over the years, it has become clear that the H558R polymorphism can either mitigate or aggravate the effects of specific mutations in *SCN5A*. For example, this polymorphism has mitigating effects on the mutations M1766L (Ye et al., 2003) and P2006A (Shinlapawittayatorn et al., 2011), but aggravating effects on the mutations G400A (Hu et al., 2007) and A572D (Tester et al., 2010).

In 2010, Gui et al. (2010a,b) demonstrated that the H558R polymorphism also affects the E161K mutation. They carried out voltage clamp experiments on HEK-293 cells transfected with E161K mutant or wild-type sodium channel α-subunit. Like Smits et al. (2005), they observed a reduction in peak I_Na_ for E161K sodium channels compared to wild-type channels, both in H558 and R558 background. In either case, this reduction was approximately 4-fold, which is more pronounced than the 2.5-fold reduction in the study by Smits et al. (2005). With a value of ≈19 mV, the positive shift in V_1/2_ of the steady-state activation curve in H558 background was also more pronounced. The steepness of this curve showed a reduction similar to that observed by Smits et al. (2005). In R558 background, the reduction in peak I_Na_ and steepness of the steady-state activation curve were both similar to those in H558 background (Gui et al., 2010b). However, the positive shift in V_1/2_ of the steady-state activation curve was less pronounced, with a value of ≈13 mV rather than ≈19 mV. However, the potentially mitigating effects of this less pronounced shift in R558 background was counteracted by a -10 mV shift in V_1/2_ of the steady-state inactivation curve, which was absent in H558 background.

To assess the mechanism by which the E161K mutation causes sinus node dysfunction as well as the potential role of the H558R polymorphism, we incorporated the mutation-induced changes in I_Na_ into the Fabbri-Severi model of a single human SA node cell (Fabbri et al., 2017). Furthermore, we incorporated these changes into the Maleckar et al. human atrial cell model (Maleckar et al., 2008, 2009) to assess the effects of the E161K mutation on atrial excitability, which may also play a role in sinus node dysfunction.

## Materials and Methods

### Implementation of the E161K Mutation and H558R Polymorphism

Effects of the heterozygous E161K mutation in *SCN5A* were implemented in the CellML code (Cuellar et al., 2003) of the Fabbri-Severi human SA nodal cell model (Fabbri et al., 2017) by scaling the fully-activated conductance of I_Na_ (g_Na_) and shifting the steady-state I_Na_ activation and/or inactivation curves, as detailed below, based on the experimental data from literature described in the Introduction and summarized in Table 1. The slight change in steepness of the steady-state activation curve was also included. These modifications were applied to half of the intrinsic I_Na_ channels, thus representing the heterozygous nature of the mutation, taking into account that a functional I_Na_ channel is built with a single Na_V_1.5 protein.

**Table 1:** Experimental data on biophysical effects of the E161K mutation in *SCN5A*.

					Steady-state activation			Steady-state inactivation		
Study	Expression system	Background	Channel type	Peak current density (nA/pF)	V_1/2_ (mV)	k (mV)	*n*	V_1/2_ (mV)	k (mV)	*n*
**Experimental Data**
Smits et al., 2005	tsA201 cells	H558	Wild-type	0.71 ± 0.11	-42.6 ± 1.4	6.7 ± 0.4	9	-89.4 ± 1.2	-4.9 ± 0.3	9
			E161K	0.28 ± 0.05^∗^	-30.7 ± 0.8^∗^	7.9 ± 0.3^∗^	13	-88.5 ± 0.9	-4.4 ± 0.1	13
Gui et al., 2010a	HEK-293 cells	H558	Wild-type	0.87 ± 0.08	-34.7 ± 0.7	6.9 ± 0.2	23	-81.4 ± 0.7	-6.4 ± 0.1	24
			E161K	0.20 ± 0.03^∗^	-14.9 ± 0.7^∗^	9.0 ± 0.1^∗^	19	-79.9 ± 0.9	-6.6 ± 0.1	21
Gui et al., 2010b	HEK-293 cells	H558	Wild-type	0.81 ± 0.08	-33.8 ± 0.7	6.7 ± 0.2	11	-80.3 ± 0.9	-6.2 ± 0.1	12
			E161K	0.19 ± 0.03^∗^	-14.7 ± 0.6^∗^	8.9 ± 0.1^∗^	11	-78.4 ± 0.8	-6.5 ± 0.2	12
		R558	Wild-type	0.92 ± 0.17	-35.5 ± 1.3	6.8 ± 0.2	9	-81.8 ± 1.0	-6.5 ± 0.1	10
			E161K	0.15 ± 0.02^∗^	-20.9 ± 1.2^∗^	8.0 ± 0.1^∗^	14	-88.8 ± 1.8^∗^	-6.7 ± 0.2	10
**Model Parameters**
Fabbri et al., 2017					-30.5	6.5		-69.8	-4.5	
Maleckar et al., 2009					-15.7	6.4		-63.6	-5.3	


*All experimental data obtained at room temperature and expressed as mean ± SEM. ^∗^P < 0.05 vs. wild-type.*

Identical changes were applied to the Maleckar et al. (2009) human atrial cell model. The latter model, which is also known as the “human atrial myocyte with new repolarization” (hAMr) model, was selected because it includes well-validated equations for the acetylcholine-activated potassium current I_K,ACh_ (Maleckar et al., 2008), thus allowing simulations over a wide range of acetylcholine levels. Action potentials were elicited with a 1 ms, ≈50% suprathreshold stimulus current at a frequency of 1 Hz. The threshold stimulus current amplitude was determined by increasing the stimulus current amplitude in 0.1 pA/pF steps until a train of 200 action potentials could successfully be elicited. To prevent slow drifts in ion concentrations, the intracellular Na^+^ and K^+^ concentrations were fixed, as were the cleft ion concentrations.

In H558 background, the E161K mutation was implemented by shifting the steady-state activation curve of the model I_Na_ by +19 mV. In R558 background, this curve was shifted by +13 mV, together with a -10 mV shift in the steady-state inactivation curve. In either background, a 50% decrease in g_Na_ was applied to arrive at an almost 4-fold decrease in peak current density of mutant I_Na_ during voltage clamp simulations. Furthermore, the slope factor of the steady-state activation curve of the model I_Na_ was increased by 20% to account for mutation-induced decrease in steepness of this curve.

### Computer Simulations

The CellML code of both models, as available from the CellML Model Repository (Lloyd et al., 2008), was edited and run in version 0.9.31.1409 of the Windows based Cellular Open Resource (COR) environment (Garny et al., 2003b). All simulations were run for a sufficiently long time, i.e., for the duration of a train of 200 action potentials, to reach steady-state behavior. Data from the final ten action potentials were used for analysis.

## Results

### Characteristics of the SA Nodal Fast Sodium Current

**FIGURE 1:**
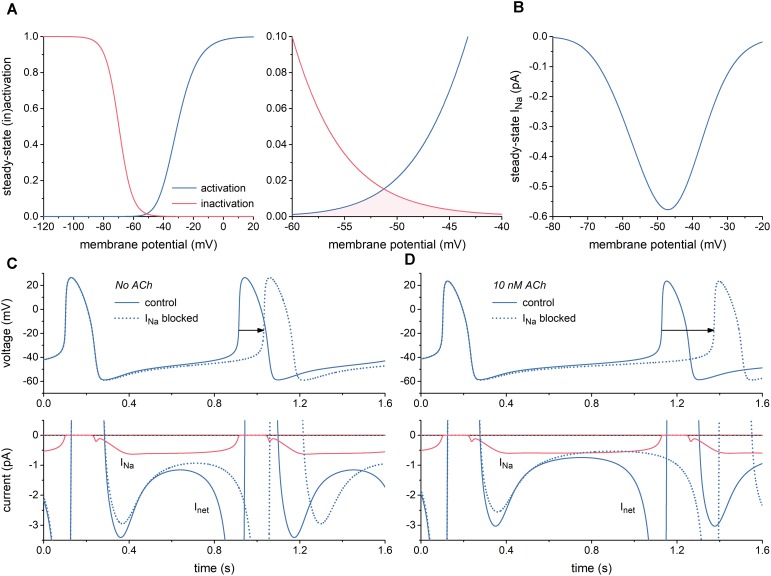
Characteristics of the fast sodium current (I_Na_) of the Fabbri-Severi model of a human SA nodal pacemaker cell. **(A)** Steady-state activation and inactivation curves (blue and red curves, respectively) of I_Na_ (left) and associated “window” of overlap (shaded area) in the voltage range between -60 and -40 mV (right). **(B)** Current–voltage relationship of steady-state I_Na_. **(C)** Effect of full block of I_Na_ in the absence of acetylcholine (ACh). Spontaneous action potentials (top) and associated net membrane current (I_net_) and I_Na_ (bottom). Horizontal arrow indicates increase in cycle length. **(D)** Effect of full block of I_Na_ during simulated administration of 10 nM ACh. Spontaneous action potentials (top) and associated I_net_ and I_Na_ (bottom). Horizontal arrow indicates increase in cycle length.

First, we characterized I_Na_ of the Fabbri-Severi model of a single human SA nodal pacemaker cell (Fabbri et al., 2017) and its effect on the spontaneous activity of this cell. Figure 1A shows the steady-state activation and inactivation curves of I_Na_ (left) and the associated “window” (area of overlap; right). The window is in the voltage range of diastolic depolarization, between -60 and -40 mV. Because of the relatively slow changes in membrane potential of the SA nodal cell and the relatively fast kinetics of I_Na_, this window is the major determinant of the course of I_Na_ during the action potential, of course in combination with the fully-activated conductance of I_Na_ (g_Na_), which amounts to 22.3 nS in the Fabbri-Severi model. The associated steady-state current-voltage relationship of I_Na_ is shown in Figure 1B.

With a value near 0.6 pA, the amplitude of I_Na_ during diastolic depolarization is small. However, this amplitude is relatively large in comparison to that of the net membrane current (I_net_) (Figure 1C, solid lines). It is therefore not surprising that full block of I_Na_ results in a 117-ms increase in cycle length from 813 to 930 ms (Figure 1C, dotted lines), corresponding with a 13% decrease in beating rate from 74 to 64 beats/min. In the simulated presence of 10 nM acetylcholine (ACh), the amplitude of I_net_ during diastolic depolarization becomes considerably smaller, whereas that of I_Na_ does barely change. Accordingly, full block of I_Na_ now results in a more prominent increase in cycle length, by 245 ms from 1027 to 1272 ms (Figure 1D), corresponding with a 19% decrease in beating rate from 58 to 47 beats/min. Of note, I_Na_ hardly affects the cycle length through a change in action potential duration.

Now that the simulations of Figure 1 had shown that changes in I_Na_ may modify the cycle length of the Fabbri-Severi model cell to a considerable extent, we first assessed the changes in peak and window I_Na_ caused by the E161K mutation in *SCN5A*, either in H558 or R558 background. Next, we tested the effects of these changes on the spontaneous pacemaker activity of the SA nodal model cell.

### Effects of the E161K Mutation in SCN5A on Biophysical Properties of I_Na_

**FIGURE 2:**
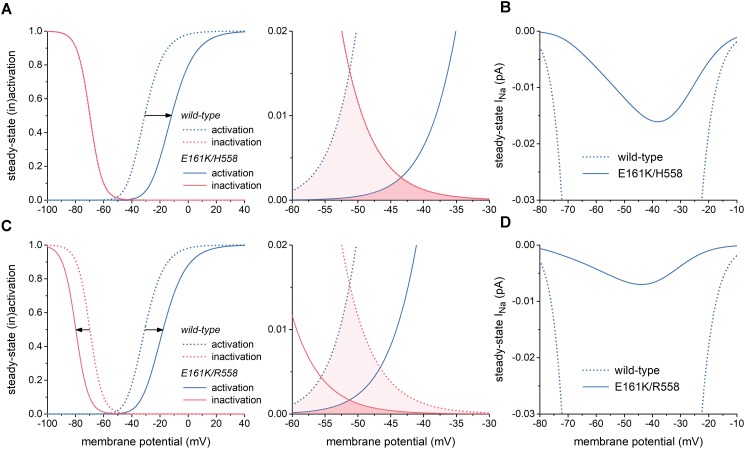
Effects of the E161K mutation in *SCN5A*, either in H558 or R558 background, on biophysical properties of the fast sodium current (I_Na_) of the Fabbri-Severi model of a human SA nodal pacemaker cell. **(A)** Shift and change in steepness of the steady-state activation curve (left) and effect on “window” (right) in H558 background. Horizontal arrow indicates the +19 mV shift. **(B)** Resulting effect on current–voltage relationship of steady-state I_Na_ in H558 background. **(C)** Shift and change in steepness of the steady-state activation curve and shift of steady-state inactivation curve (left) and effect on “window” (right) in R558 background. Horizontal arrows indicate the +13 and -10 mV shifts. **(D)** Resulting effect on current–voltage relationship of steady-state I_Na_ in R558 background.

The left panel of Figure 2A illustrates the changes in the steady-state I_Na_ activation and inactivation curves due to the E161K mutation in H558 background. These changes are limited to a +19 mV shift in the steady-state I_Na_ activation curve and a slight decrease in steepness of this curve, which is poorly discernible in Figure 2A. As a result, the window of overlap is significantly reduced and shifted to less negative values of membrane potential, now roughly ranging from -50 to -30 mV (Figure 2A, right) and thus limiting the contribution of I_Na_ to diastolic depolarization. This is reflected in the current–voltage relationship of steady-state I_Na_ (Figure 2B, solid line), which is strongly reduced in comparison with wild-type I_Na_.

In R558 background, the shift in the steady-state I_Na_ activation curve is less pronounced (+13 vs. +19 mV), but accompanied by a -10 mV shift in the steady-state inactivation curve (Figure 2C, left). As a result, the window of overlap is smaller than in H558 background (Figure 2C, right). However, it better fits with the voltage range of diastolic depolarization. Yet, the steady-state I_Na_ in this voltage range is even more reduced in comparison with wild-type I_Na_ than in H558 background (Figure 2D, solid line).

### Effects of the E161K Mutation in SCN5A on I_Na_ in Voltage Clamp Experiments

**FIGURE 3:**
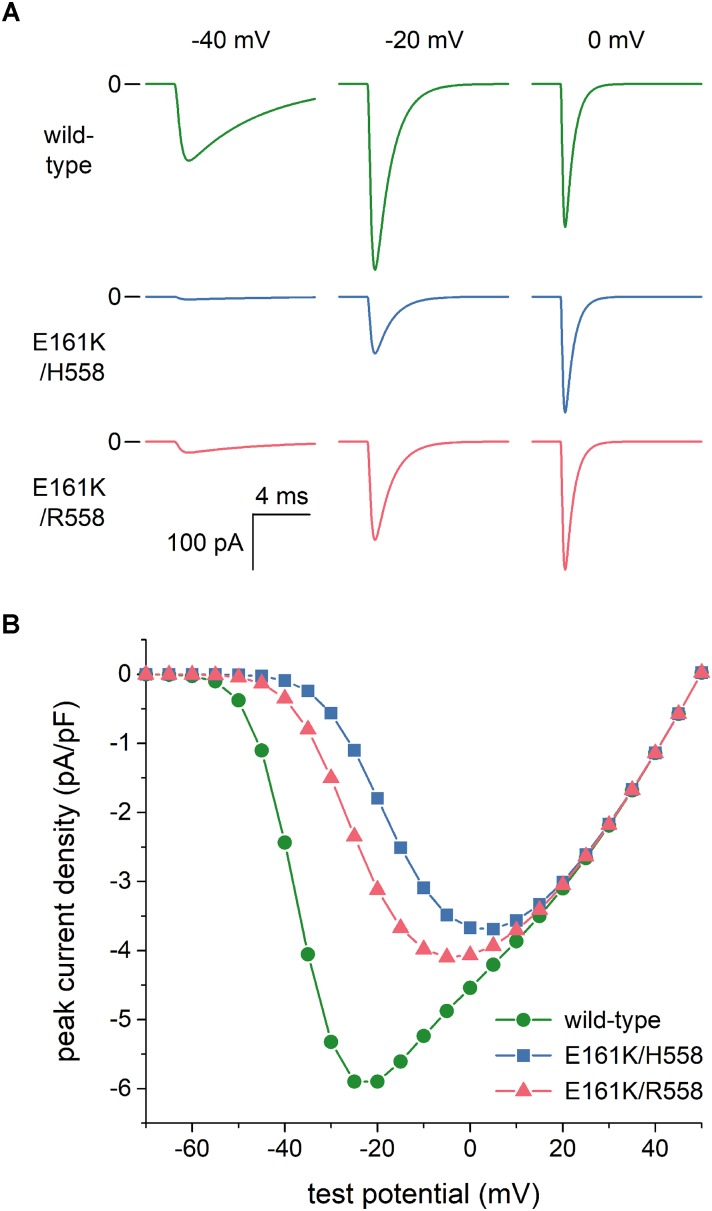
Effects of the E161K mutation in *SCN5A*, either in H558 or R558 background, on the fast sodium current (I_Na_) of the Fabbri-Severi model of a human SA nodal pacemaker cell during simulated voltage clamp experiments. **(A)** Control (“wild-type”) and mutant I_Na_ during voltage clamp steps from a holding potential of -120 mV to test potentials of -40, -20, and 0 mV. **(B)** Current–voltage relationship of peak I_Na_, as derived from these simulated voltage clamp experiments.

As set out in the Introduction, voltage clamp experiments on *SCN5A* channels expressed in tsA201 and HEK-293 cells (Smits et al., 2005; Gui et al., 2010a,b) had revealed that the E161K mutation induces a 2.5- to 4-fold decrease in I_Na_ peak current density, both in H558 and R558 background. To assess the extent to which this reduction is due to the changes in the I_Na_ steady-state activation and inactivation curves *per se*, we reconstructed current traces in response to voltage clamp steps from a holding potential of -120 mV to test potentials ranging from -100 to +50 mV. Figure 3A shows examples of wild-type and mutant current traces at test potentials of -40, -20, and 0 mV. These already demonstrate that the peak current density of the wild-type channels is higher than that of mutant channels, despite the identical value of g_Na_ used in the voltage clamp simulations. Figure 3B summarizes the simulation data. The changes in the steady-state activation and inactivation curves *per se* reduce the peak current density by ≈35%. To arrive at an almost 4-fold decrease in I_Na_ peak current density, in line with the experimental observations, we reduced the mutant g_Na_ by a factor of 2 in our further simulations. Thus we split the original g_Na_ of 22.3 nS of the Fabbri-Severi model cell into 11.15 nS for the wild-type I_Na_ channels and 5.575 nS for the E161K mutant I_Na_ channels, in either background.

### Effects of the E161K Mutation on SA Nodal Pacemaker Activity

**FIGURE 4:**
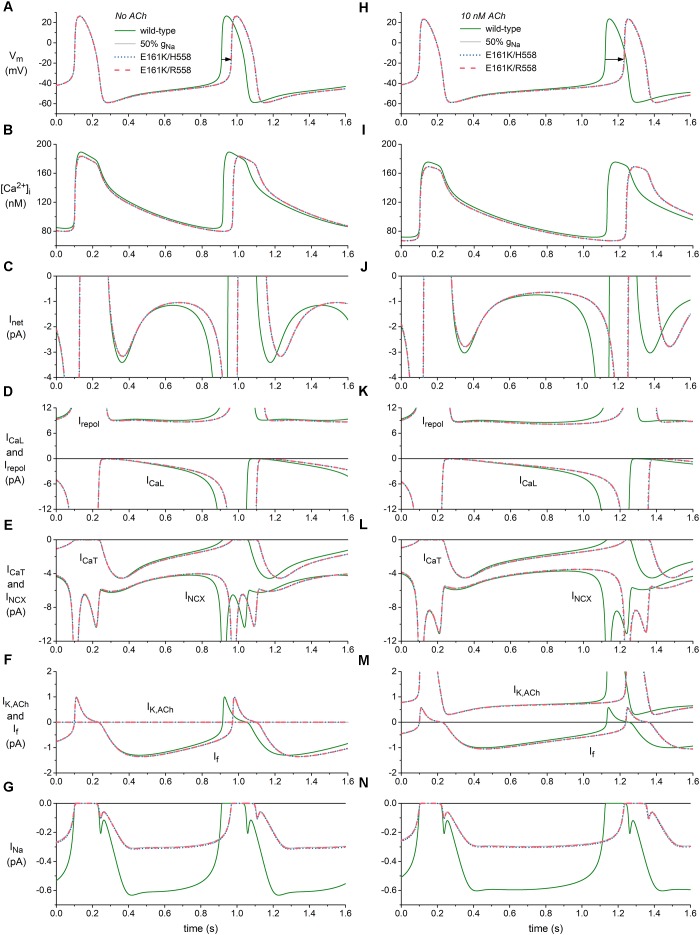
Effects of the E161K mutation in *SCN5A*, either in H558 or R558 background, on the spontaneous activity of the Fabbri-Severi model of a human SA nodal pacemaker cell. **(A)** Membrane potential (V_m_), and associated **(B)** intracellular calcium concentration ([Ca^2+^]_i_), **(C)** net membrane current (I_net_), **(D)** L-type calcium current (I_CaL_) and total repolarizing current (I_repol_), **(E)** T-type calcium current (I_CaT_) and sodium-calcium exchange current (I_NCX_), **(F)** ACh-activated potassium current (I_K,ACh_) and hyperpolarization-activated “funny current” (I_f_), and **(G)** fast sodium current (I_Na_) in the absence of ACh. **(H)** V_m_, **(I)** [Ca^2+^]_i_, **(J)** I_net_, **(K)** I_CaL_ and I_repol_, **(L)** I_CaT_ and I_NCX_, **(M)** I_K,ACh_, and I_f_, and **(N)** I_Na_ during simulated administration of 10 nM ACh. Solid gray lines show the effect of a 50% reduction in fully-activated conductance of I_Na_ (g_Na_). Horizontal arrows indicate increase in cycle length. I_repol_ consists of I_K,ACh_, the rapid delayed rectifier potassium current I_Kr_, the slow delayed rectifier potassium current I_Ks_, the ultrarapid delayed rectifier potassium current I_Kur_, the transient outward potassium current I_to_, and the sodium-potassium pump current I_NaK_.

We applied the mutation-induced changes in I_Na_, i.e., the shifts in steady-state activation and inactivation curves, the reduction in steepness of the steady-state activation curve, and the reduction in fully-activated conductance, to half of the I_Na_ channels in the Fabbri-Severi model to assess the effects of the heterozygous E161K mutation on the spontaneous pacemaker activity of a human SA nodal cell. Figures 4A–G, shows the effects on the action potential (Figure 4A), intracellular calcium concentration (Figure 4B) and underlying membrane currents (Figures 4C–G) under control conditions (no ACh), whereas the corresponding simulation data in the presence of 10 nM ACh are shown in Figures 4H–N. The simulated administration of ACh to the Fabbri-Severi model cell does not only activate the acetylcholine-activated potassium current I_K,ACh_, which is zero under control conditions, but also reduces the hyperpolarization-activated “funny current” I_f_ (through a -4.95 mV shift in its voltage dependence of activation), the L-type calcium current I_CaL_ (through a 3.1% decrease in its maximal conductance), and the rate of Ca^2+^ uptake into the sarcoplasmic reticulum (SR) by the SERCA pump (through a 7.0% decrease in its maximum activity). Of note, the changes in I_CaL_ and Ca^2+^ uptake rate have only marginal effects at this level of ACh (Fabbri et al., 2017).

The main effect of the application of ACh is a prolongation of the basic cycle length from 813 to 1027 ms (Figures 4A,H, wild-type traces), which is associated with a decrease in the intracellular calcium concentration [Ca^2+^]_i_ (Figures 4B,I, wild-type traces), and sodium-calcium exchange current I_NCX_ during diastolic depolarization (Figures 4E,L, wild-type traces). I_f_ is also reduced (Figures 4F,M), as a result of the direct effect of ACh on its voltage dependence of activation. The remaining inward currents, i.e., the L-type calcium current I_CaL_ (Figures 4D,K), T-type calcium current I_CaT_ (Figures 4E,L) and I_Na_ (Figures 4G,N), are not largely affected. The total repolarizing current I_repol_ is also hardly affected, with an almost constant value of ≈9 pA during diastolic depolarization (Figures 4D,K). This is because it includes the ACh-activated I_K,ACh_, which is also shown separately (Figures 4F,M), in addition to the rapid delayed rectifier potassium current I_Kr_, the slow delayed rectifier potassium current I_Ks_, the ultrarapid delayed rectifier potassium current I_Kur_, the transient outward potassium current I_to_, and the sodium-potassium pump current I_NaK_, which are not shown separately in Figure 4. The reduction in the “pacemaker currents” I_f_ and I_NCX_ (Lakatta and DiFrancesco, 2009), in combination with the minor changes in other inward currents as well as I_repol_, result in the reduction in the net membrane current I_net_ (Figures 4C,J) that underlies the observed increase in cycle length.

In absence of ACh, the mutation-induced reduction in I_Na_ (Figure 4G) causes a reduction in I_net_, which becomes most prominent during the second half of diastolic depolarization (Figure 4C) and in turn results in a prolongation of the cycle length by 53 and 54 ms in H558 and R558 background, respectively (Figure 4A, horizontal arrow), from the basic cycle length of 813 ms, corresponding with a 6% decrease in beating rate from 74 to 69 beats/min in either background. Simulations with the wild-type model in which g_Na_ is reduced by 50% (labeled “50% g_Na_”), thus simply blocking 50% of the channels, result in a cycle length prolongation of 55 ms and traces that are barely distinguishable from the mutant ones, thus demonstrating that the contribution of the mutant component of I_Na_ to total I_Na_ is almost zero.

In the simulated presence of 10 nM ACh, the effects of the mutation are more pronounced, as reflected by the prolongation of the cycle length by 104 ms in H558 background and 105 ms in R558 background (Figure 4H, horizontal arrow), from the basic cycle length of 1027 ms, corresponding with a 9% decrease in beating rate from 58 to 53 beats/min in either background. Again, the contribution of the mutant component of I_Na_ to total I_Na_ is almost zero, as demonstrated by the highly similar prolongation of cycle length, by 107 ms, in case of 50% g_Na_. The more pronounced effect of the highly similar reduction in I_Na_ in the simulated presence of 10 nM ACh (Figures 4G,N) can be explained by its occurrence in the setting of a smaller I_net_ during diastolic depolarization (Figures 4C,J).

**FIGURE 5:**
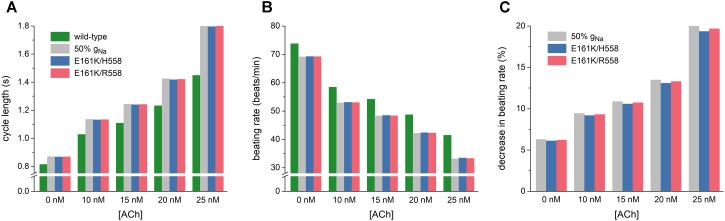
Bradycardic effects of the E161K mutation in *SCN5A*, either in H558 or R558 background, on the Fabbri-Severi model of a human SA nodal pacemaker cell at different levels of ACh (0–25 nM). **(A)** Cycle length. **(B)** Associated beating rate. **(C)** Percent decrease in beating rate relative to wild-type. Solid gray bars show the effect of a 50% reduction in fully-activated conductance of I_Na_ (g_Na_). Note the breaks in the vertical axes of **(A,B)**.

As illustrated in Figure 5A, the mutation-induced increase in cycle length increases with increasing levels of ACh. At 25 nM, this increase amounts to 347 and 355 ms in H558 and R558 background, respectively, from a basic cycle length of 1448 ms, slightly less than the increase of 361 ms in case of 50% g_Na_. In terms of beating rate (Figure 5B), an ACh level of 25 nM results in a rate of 41 beats/min in case of wild-type sodium channels and 33 beats/min in case of the heterozygous E161K mutation, in either background. The percent decrease in beating rate relative to wild type is shown in Figure 5C. In absence of ACh (Figure 5C, leftmost bars), the E161K mutation results in a 6.1% decrease in beating rate in H558 background and 6.2% decrease in R558 background. This percentage increases with increasing levels of ACh. In the simulated presence of 10 nM ACh, the decrease in beating rate amounts to 9.2 and 9.3%, respectively. At an ACh level of 25 nM (Figure 5C, rightmost bars), the mutation-induced decrease in beating rate is almost 20%, with values of 19.3% in H558 background and 19.7% in R558 background.

### Effects of the E161K Mutation on Atrial Excitability

Sinus node dysfunction may also be related to changes in atrial excitability, potentially leading to sinus node exit block or atrial conduction block. Therefore, we assessed the effects of the E161K mutation on atrial excitability using the Maleckar et al. human atrial cell model (Maleckar et al., 2009), which we used in combination with its well-validated equations for I_K,ACh_ (Maleckar et al., 2008). Changes in I_Na_, simulating a heterozygous mutation in *SCN5A*, were implemented as in the Fabbri-Severi model. Of note, the effect of ACh on the Maleckar et al. model cell is limited to the activation of I_K,ACh_.

**FIGURE 6:**
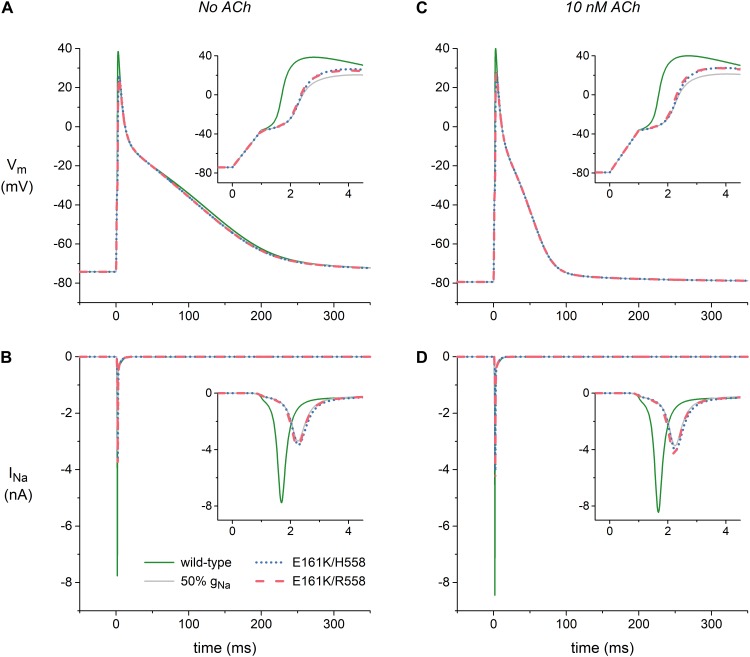
Effects of the E161K mutation in *SCN5A*, either in H558 or R558 background, on the action potential and fast sodium current (I_Na_) of the Maleckar et al. model of a human atrial myocyte. **(A)** Action potentials elicited at 1 Hz and **(B)** associated fast sodium current (I_Na_) in the absence of ACh. **(C)** Action potentials elicited at 1 Hz and **(D)** associated I_Na_ during simulated administration of 10 nM ACh. Insets show membrane potential (V_m_) and I_Na_ on an expanded time scale. Solid gray lines show the effect of a 50% reduction in fully-activated conductance of I_Na_ (g_Na_).

Figure 6A shows atrial action potentials elicited at 1 Hz with a stimulus current of 1 ms duration under control conditions (no ACh). Both variants of the E161K mutation result in a reduction in the action potential overshoot and a less rapid activation, indicative of a decrease in capacitive current of the atrial cell (Figure 6A, inset). The associated I_Na_, which is shown in Figure 6B, is approximately halved or – because the slower activation allows a larger fraction of the channels to inactivate during the activation process – even more than halved. In the simulated presence of 10 nM ACh, the resting membrane potential becomes more negative with a value of -79 mV vs. -74 mV (Figure 6C), which results in a larger I_Na_ because less sodium channels are inactivated at this more negative resting potential (Figure 6D). This does, however, not imply that action potential generation is facilitated in presence of ACh since the distance to action potential threshold is increased and an additional outward current, i.e., I_K,ACh_, is activated. Again, activation is slowed and the underlying I_Na_ is approximately halved (Figures 6C,D, insets).

**FIGURE 7:**
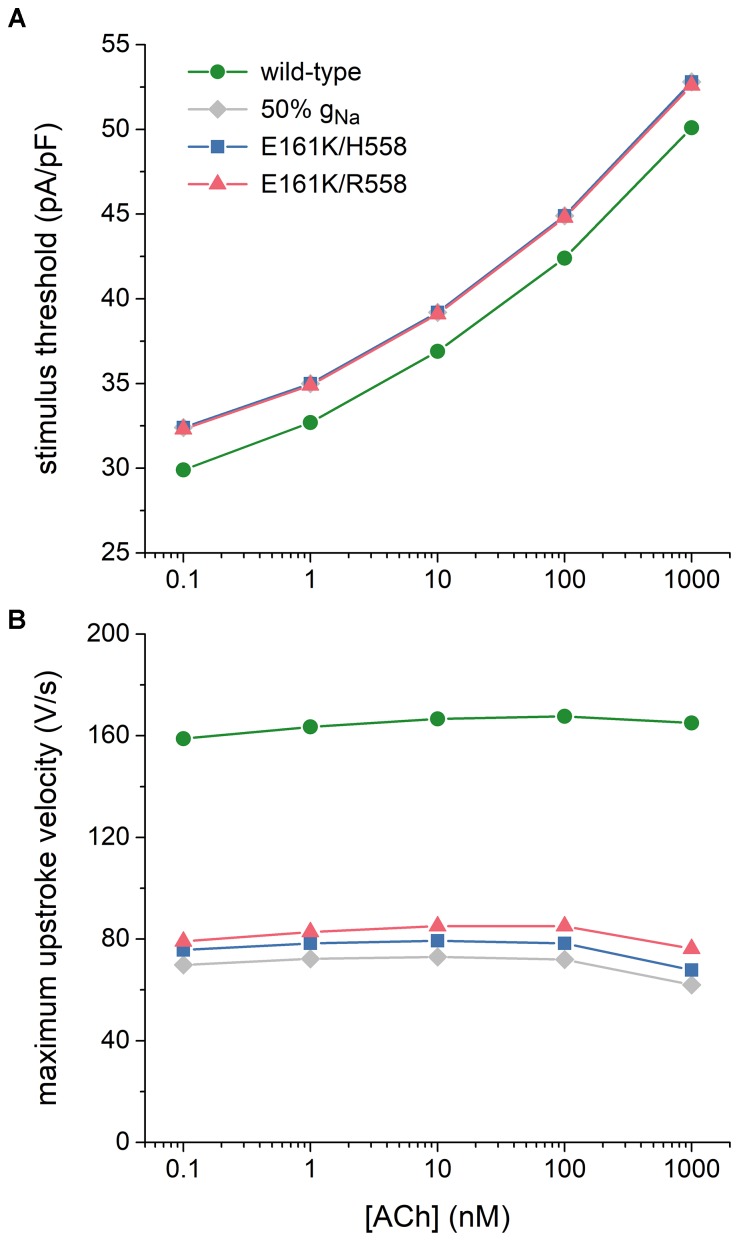
Effects of the E161K mutation in *SCN5A*, either in H558 or R558 background, on the excitability of the Maleckar et al. model of a human atrial myocyte. **(A)** Threshold stimulus current amplitude for a stimulus of 1 ms duration and **(B)** maximum upstroke velocity at ACh concentrations ranging from 0.1 nM to 1 μM. Gray diamonds show the effect of a 50% reduction in fully-activated conductance of I_Na_ (g_Na_). Note the logarithmic abscissa scale.

Atrial excitability was characterized, over a wide range of concentrations of ACh, by determining the threshold stimulus current of the atrial cell as well as its maximum upstroke velocity, as a direct measure of the capacitive current. Threshold stimulus current was determined with 1 ms stimuli of increasing amplitude that were applied at a frequency of 1 Hz. Results are shown in Figure 7A. The threshold increases with increasing levels of ACh and is 5–8% higher in case of the E161K mutation. Although barely visible in Figure 7A, the threshold in R558 background, with the smaller shift of the steady-state activation curve, is smaller than in H558 background.

Maximum upstroke velocity was determined by eliciting action potentials with a 1 ms, ≈50% suprathreshold stimulus current at a frequency of 1 Hz. As shown in Figure 7B, it is not strongly dependent on the level of ACh. Upstroke velocity is approximately halved, or even more than halved, in case of the E161K mutation. As for the threshold stimulus current, the mutation in R558 background is associated with (slightly) less strong effects than in H558 background. In contrast with the SA nodal cell, the mutant component of I_Na_ is not effectively zero, as revealed by the larger I_Na_ (Figures 6B,D, insets) and larger maximum upstroke velocity (Figure 7B) in comparison with the 50% g_Na_ simulations.

## Discussion

### General Discussion

Our simulations demonstrate that the E161K mutation in *SCN5A* hampers both impulse generation and impulse propagation through its effects on the electrophysiological properties of human SA nodal and atrial cells. Generation of the SA nodal action potential is hampered by the strong decrease in I_Na_ during diastolic depolarization and the associated decrease in I_net_, in particular in presence of ACh (Figures 4, 5). Impulse propagation, as comprehensively reviewed by Kléber and Rudy (2004), is hampered by the current-to-load mismatch that results from, on the one hand, the increase in threshold stimulus current and, on the other hand, the decrease in capacitive current of the atrial cell (Figures 6, 7). The highly common H558R polymorphism in *SCN5A* does not have a major effect on the outcome of the simulations, despite its effects on the biophysical properties of the E161K mutant channels (Figures 2, 3).

The E161K mutation has been subject of simulations since its discovery in 2005 (Smits et al., 2005). Smits et al. (2005) showed that the E161K mutation impairs conduction in linear strands of atrial and ventricular cells, using the human atrial and ventricular cell models by Courtemanche et al. (1998) and Priebe and Beuckelmann (1998), respectively. Simulations were based on the observations by Smits et al. (2005) on E161K channels expressed in tsA201 cells. Mutation effects were relatively mild in comparison to later observations by Gui et al. (2010a,b) and obtained in H558 background. Smits et al. (2005) also carried out simulations on SA nodal cells, using the model of a peripheral rabbit SA nodal cell by Zhang et al. (2000), including equations for I_K,ACh_ (Zhang et al., 2002). Despite the largely different time course of the human SA nodal action potential, with a much smaller I_Na_, a much longer diastolic phase, and a significantly less negative maximum diastolic potential, the main findings of the present study are similar to those of Smits et al. (2005) regarding the SA nodal action potential: the E161K mutation causes a decrease in diastolic depolarization rate that results in an increase in cycle length, which is much more pronounced in presence of ACh.

Butters et al. (2010) studied the effects of several mutations in *SCN5A*, including E161K, on cardiac pacemaking in a two-dimensional model of sino-atrial tissue based on a reconstruction of a single slice of the rabbit right atrium, using modified versions of the single cell models of rabbit SA nodal and atrial cells by Zhang et al. (2000, 2002) and Lindblad et al. (1996), respectively. Their simulations of the E161K mutation were again based on the observations by Smits et al. (2005). The mutation slowed down pacemaking and this effect was more pronounced in the presence of ACh. Furthermore, a conduction block in the direction toward the atrial septum occurred in the absence of ACh, while conduction in the direction toward the crista terminalis was sustained. With 15 nM ACh, exit block occurred in both directions and the SA node was unable to drive the surrounding atrium.

In the present study, we carried out simulations with the use of comprehensive well-validated human single cell models and implemented effects of the E161K mutation based on the experimental data by Gui et al. (2010a,b). Furthermore, we assessed the role of the highly common H558R polymorphism, also based on the experimental data by Gui et al. (2010a,b). We focused on cellular mechanisms and refrained from using two- or even three-dimensional models of sino-atrial interaction, with their coupling interactions between heterogeneous cells, because results of such simulations are critically dependent on the exact geometry and cell distribution (Garny et al., 2003a), which have not been fully elucidated.

Our simulations show that the effects of the E161K mutation are so large, both in H558 and R558 background, that the contribution of mutant I_Na_ to total I_Na_ of the SA nodal cell is effectively zero. Accordingly, it is not surprising that E161K mutation carriers show sinus node dysfunction as do carriers of several mutations in *SCN5A* that are associated with a complete or almost complete loss of function of the mutant channels, as reviewed by Lei et al. (2007, 2008) and Remme et al. (2008). In many cases, the sinus node dysfunction shows a reduced penetrance, as for the E161K mutation, where 8 out of 14 mutation carriers show the disease. Reduced penetrance is a common finding in primary electrical diseases, including those associated with mutations in *SCN5A* (Remme, 2013; Robyns et al., 2014). Several factors, including co-inherited genetic variants and alternative splicing sites, may play a role. However, our simulations suggest that it is highly unlikely that the common H558R polymorphism can explain the reduced penetrance of the E161K mutation.

### Limitations

Unfortunately, experimental data on the effects of the E161K mutation are limited to voltage clamp data from mutant *SCN5A* channels in mammalian expression systems, viz. tsA201 and HEK-293 cells, obtained at room temperature and in the presence (Smits et al., 2005) or absence (Gui et al., 2010a,b) of a wild-type β-subunit. There are some quantitative differences in the experimental data, potentially due to differences in cell type or presence of β-subunit, with a more severe pattern in the data of Gui et al. (2010a,b), with a +19 mV shift of the steady-state activation curve in H558 background vs. the +11.9 mV shift reported by Smits et al. (2005) and a 4-fold vs. 2.5-fold reduction in peak current density. However, the latter difference may partly be explained by the larger shift in the steady-state activation curve. In our simulations, we have assumed that the experimentally observed effects of the mutation in mammalian expression systems at room temperature also hold for cardiac cells at the physiological temperature of 37°C. We cannot rule out that recordings at a more close-to-physiological temperature would have revealed additional effects of the E161K mutation, as has been the case for several other BrS-related mutations in *SCN5A* expressed in mammalian cell lines, like T1620M and Y1795H (Dumaine et al., 1999; Rivolta et al., 2001). Furthermore, we have to keep in mind that the cardiac sodium channel is part of a macromolecular complex that does not only comprise the α- and β-subunits, but also several other proteins that may regulate channel activity, such as ankyrin, caveolin and syntrophin, and may act differently in case of mutant channels (Ruan et al., 2009). Thus, it is uncertain to which extent the experimental data on the effects of the E161K mutation acquired in mammalian expression systems represent the mutant-induced changes that occur in human cardiac cells.

In our simulations, it is assumed that the Fabbri-Severi model of a human SA nodal cell (Fabbri et al., 2017) and the Maleckar et al. model of a human atrial cell (Maleckar et al., 2009) are fully representative of the biophysical properties of the corresponding real cells. However, experimental data on the biophysical properties of cardiac cells, and human cells in particular, are often incomplete or obtained under non-physiological conditions. Functional data on I_Na_ in human SA nodal cells, for example, are limited to accidental observations by Verkerk et al. (2009) when performing voltage clamp experiments on single human SA nodal pacemaker cells to record I_f_. Accordingly, I_Na_ was included in the Fabbri-Severi model, but simply adopted from its parent model, i.e., the model of a rabbit SA nodal cell by Severi et al. (2012), who, in turn, used the equations of the SA nodal cell model by Noble et al. (1989). The latter equations were adopted from the model of SA nodal cell electrical activity by Noble and Noble (1984), who based their equations on the model of cardiac electrical activity by DiFrancesco and Noble (1985). The description of I_Na_ in the latter model is essentially that of Hodgkin and Huxley (1952), but with modifications based on data from experiments on rabbit cardiac Purkinje fibers at 10–26°C (Colatsky, 1980) and from isolated rat ventricular myocytes at 20–22°C (Brown et al., 1981). Activation and inactivation curves were shifted along the voltage axis to account for their observed temperature dependence, whereas rate constants were scaled to arrive at time constant values near the experimentally observed ones. The I_Na_ equations in the Maleckar et al. (2009) model are adopted from the model of an adult human atrial cell by Nygren et al. (1998), who used the voltage clamp data of Sakakibara et al. (1992) on I_Na_ in isolated human atrial myocytes at 17 ± 1°C to construct their model equations. Activation and inactivation curves were shifted in the positive direction along the voltage axis to reach a realistic activation threshold and stable resting potential. The time constants of activation and inactivation are very similar to those of the rabbit atrial cell model of Lindblad et al. (1996), who based their mathematical description of I_Na_ on the voltage clamp data of Wendt et al. (1992), acquired from cultured rabbit atrial myocytes at 17°C, and I_Na_ of the DiFrancesco-Noble model (DiFrancesco and Noble, 1985). Activation kinetics of I_Na_ were modified from those of the DiFrancesco-Noble model with the use of a Q_10_ of 1.7.

In our simulations, it is also assumed that mutation effects are limited to half of the intrinsic I_Na_ channels, thus representing the heterozygous nature of the mutation, taking into account that a functional I_Na_ channel is built with a single Na_V_1.5 protein. This assumption is challenged by recent experimental findings of Clatot et al. (2018), who demonstrated that wild-type and mutant Na_V_1.5 proteins can form dimers, enabling coupled gating of wild-type and mutant Na_V_1.5 sodium channels that can be responsible for a dominant negative effect of the mutation. Thus, the cellular effects of the E161K mutation could be more severe than anticipated.

The action of ACh in the Fabbri-Severi model is, apart from the activation of I_K,ACh_, limited to suppression of I_f_, I_CaL_, and Ca^2+^ uptake into the SR, thus simplifying the intracellular SR-based calcium clock signaling cascade through adenylyl cyclase, cyclic AMP and protein kinase A (Maltsev et al., 2014; Behar et al., 2016). This cascade is important for a detailed understanding of the action of ACh. Yet, in the present study, where the focus is on the effects of the E161K mutation in *SCN5A* and possible effects of the H558R polymorphism, it is sufficient that the suppression by ACh of I_f_, I_CaL_, and Ca^2+^ uptake into the SR, and that of I_NCX_ through the reduced intracellular calcium level, are included, in addition to the activation of I_K,ACh_.

One should be careful in the interpretation of experimental data, not only because of the above considerations, but also because changes in biophysical parameters are not necessarily independent. This is nicely demonstrated by the decrease in peak current density shown in Figure 3. Such decrease is often interpreted as a reduction in the number of channels or a reduction in their single conductance. In this case, however, the E161K mutant-induced changes in steady-state activation and inactivation curves *per se* reduce the peak current density by ≈35%. This effect may, at least partly, explain the apparent discrepancy between the ≈30% decrease in cell surface expression of E161K/H558 channels in HEK-293 cells and the > 70% decrease in peak current density reported by Gui et al. (2010a). Similarly, an apparently slower inactivation of E161K/H558 channels at membrane potentials ranging from -40 to 0 mV can, at least partly, be explained by the +19 mV shift in their steady-state activation curve (Gui et al., 2010a). Because experimentally observed changes in time constants of (fast) inactivation or recovery from inactivation, if any, are already small, no attempts were made to include these changes in the simulations of the present study.

## Conclusion

We conclude that the experimentally identified mutation-induced changes in I_Na_ can explain the clinically observed sinus bradycardia and potentially the occasional exit block. Furthermore, we conclude that the common H558R polymorphism does not significantly alter the effects of the E161K mutation and can thus not explain the reduced penetrance of the E161K mutation.

## Data Availability Statement

The raw data supporting the conclusions of this manuscript will be made available by the author, without undue reservation, to any qualified researcher.

## Author Contributions

RW designed the experiments, acquired, analyzed and interpreted the data, and drafted, edited, and approved the manuscript.

## Conflict of Interest Statement

The author declares that the research was conducted in the absence of any commercial or financial relationships that could be construed as a potential conflict of interest.
